# Development and Challenges in Animal Tuberculosis Vaccination

**DOI:** 10.3390/pathogens9060472

**Published:** 2020-06-15

**Authors:** Ana Balseiro, Jobin Thomas, Christian Gortázar, María A. Risalde

**Affiliations:** 1 Departamento de Sanidad Animal, Facultad de Veterinaria, Universidad de León, 24071 León, Spain; 2 Departamento de Sanidad Animal, Instituto de Ganadería de Montaña (CSIC-Universidad de León), Finca Marzanas, Grulleros, 24346 León, Spain; 3 SaBio-Instituto de Investigación en Recursos Cinegéticos IREC (UCLM-CSIC-JCCM), Universidad de Castilla-la Mancha (UCLM), 13071 Ciudad Real, Spain; jobss2K6@gmail.com (J.T.); christian.gortazar@uclm.es (C.G.); 4 Indian Council of Agricultural Research (ICAR), New Delhi 110001, India; 5 Departamento de Anatomía y Anatomía Patológica Comparadas y Toxicología. Facultad de Veterinaria. Universidad de Córdoba (UCO), 14014 Córdoba, Spain; v12rimom@uco.es; 6 Unidad de Enfermedades Infecciosas, Grupo de Virología Clínica y Zoonosis, Instituto Maimónides de Investigación Biomédica de Córdoba (IMIBIC), Hospital Reina Sofía, Universidad de Córdoba (UCO), 14004 Córdoba, Spain

**Keywords:** vaccination, tuberculosis, Bacillus Calmette–Guérin (BCG), heat-inactivated *Mycobacterium bovis* vaccine (HIMB), domestic animal, wildlife

## Abstract

Vaccination with Bacillus Calmette–Guérin (BCG) constituted a major advance in the prevention of human tuberculosis (TB) in the beginning of the past century. BCG has also a clear potential for use in animals and, in particular, in the main domestic species subjected to TB control programs, cattle. Nowadays, the use of BCG vaccination against TB in cattle is not permitted by European Union legislation because BCG can induce a cellular immune response producing diagnostic interference in the eradication programs based on tuberculin single and comparative intradermal tests imposed worldwide. In this review, we recall the history of TB vaccination as well as different vaccine trials and the response to vaccination in both domestic and wild animals. Promising potential inactivated vaccines are also reviewed. Research studies are mainly focused to improve vaccine efficacy, and at the same time to ensure its easy administration, safety and stability in the environment. Great challenges remain, particularly in terms of vaccine candidates and also in the acceptance of vaccination. Vaccination should be included in a strategic plan for integrated control of TB under a “one health” perspective, which also includes other measures such as improved biosafety on farms to avoid or decrease contact between domestic and wild animals or control of wildlife reservoirs to avoid overabundance that may favor infection maintenance.

## 1. History and Evolution of Tuberculosis Vaccination

Tuberculosis (TB) is a contagious and chronic infectious disease, caused by *Mycobacterium tuberculosis* complex (MTC) bacteria that has always been a continuous challenge over the course of human and animal history because of its severe sanitary, social and economic implications. The disease affects both domestic and wild animals worldwide [1]. Tuberculosis was one of the first pathologic entities in which the role of a bacterium was demonstrated as a cause of disease and, therefore, had a substantial contribution to the consolidation of the microbial theory of the disease [1]. The scientist Robert Koch isolated the tubercle bacillus and presented this great result to the society of physiology in Berlin on 24 March 1882. This advance in the knowledge of the etiology of TB, together with the empirical observation that some individuals who overcame the disease did not suffer it again, provided the basis for initiating the scientific design of vaccines. This was a major landmark and revolution in the human medicine through which the history of human population changed [2].

In the decades following the discovery of TB etiological agent, efforts to develop a vaccine to protect against TB began immediately and culminated in the discovery of the live Bacillus Calmette–Guérin (BCG) vaccine [3]. BCG was based on attenuation by successive in vitro passages of a culture of *M. bovis*, during the years 1908–1921, carried out by Calmette and Guérin at the Pasteur Institute of Lille [4]. At that time, it constituted the main medical tool for the control of human TB. Only a few decades following the distribution of BCG stocks to vaccine production laboratories worldwide, different BCG producers recognized that there were variants of BCG; one explanation was the variability in passaging conditions in the different laboratories [5]. The lyophilization of stable BCG products was achieved in the 1950s and 1960s, but considerable evolution of the different BCG strains had already taken place. The application of novel research methodologies has revealed transcriptomic, genomic and proteomic differences among BCG strains. These molecular differences of BCG strains are in part accounted for *in vitro* phenotypic differences, such as their variable secretion of antigenic proteins [5]. Currently, the most used strains in the world are BCG Danish 1331, BCG Pasteur 1173 P2, BCG Glaxo 107, BCG Tokyo 172-1, BCG Russia-I r and BCG Brazil. The only vaccine licensed in Europe for humans is BCG Danish 1331 (Pfizer, New York, NY, USA). It consists of a lyophilized of *M. bovis* (spoligotype (SB) 120) at a concentration of 1 mg/mL (2–8 × 10^6^ colony forming units (CFU)) and it is administered intradermally (WHO, 2018).

BCG had also a clear potential for use in animals and, in particular, in the main domestic TB reservoir, cattle [6]. Nowadays, the use of BCG vaccination against *M. bovis* in cattle is not permitted by European Union legislation because BCG can induce a cellular immune response producing diagnostic interference in the eradication programs based on tuberculin single (SIT) and comparative (SCITT) intradermal tests imposed worldwide [7]. To date, due to the failure in eradicating bovine TB after decades since eradication programs began in Europe in the 80s of the last century, the medical-veterinary community is rethinking whether a vaccination strategy used with scientific rigor should be a tool for the fight against TB [8,9,10]. The main issue is the lack of availability of diagnostic tests to differentiate vaccinated from infected animals, namely DIVA tests [11]. In countries with control strategies based on test and cull, DIVA tests would be necessary if vaccination with BCG or other mycobacterial-based vaccines were applied [12]. Inactivated vaccines such as heat-inactivated *Mycobacterium bovis* vaccine (HIMB) orally administered may solve this problem as its interference with diagnostic tests is minimal [13].

One specific requirement for using a vaccine in wildlife is that it should be administered by baits (oral route). In this sense, there are challenges associated with the development of a live oral BCG vaccine in field conditions. Those include that survival of BCG in baits should be maintained until deployment in the field and its uptake by wildlife, and also issues arising from release of a live vaccine into the nature. Inactivated vaccines are expected to be more stable in baits (i.e., under high environmental temperatures) and safer as they are based on dead bacteria [14]. Recent studies have begun to address these concerns by demonstrating that oral vaccination with a HIMB successfully protects captive wild boar (*Sus scrofa*) [15], Molokai-origin wild pigs [16], red deer (*Cervus elaphus*) [17] and badgers (*Meles meles*) [18] against progressive disease. Field studies have also shown protective effect of oral HIMB vaccination in piglets against TB in endemic free-ranging wild boar populations [14].

In this study (i) we present an overview of the scientific knowledge on animal TB vaccination and (ii) we discuss its use in upcoming programs in order to approach a future without TB in domestic animals and wildlife.

## 2. Types of Vaccines

### 2.1. Live Attenuated BCG

Vaccination against TB started in cattle in the early 1990s by using BCG. The efficacy of this vaccine has been validated in many experimental laboratory and field trials conducted in domestic and wild animals which included different strains, routes, doses and routes for virulent *M. bovis* challenge [19,20,21,22]. Some of these studies included cattle [23], goats [24], sheep [25], wild boar [26], white-tailed deer (*Odocoileus virginianus*) [27], red deer [28], possums (*Trichosurus vulpecula*) [29] and badgers [11,18] with varying levels of protection. However, BCG raises the problem of interference with diagnostic tests [7,30] and its stability in natural conditions is uncertain [15], as well as the possible survival in the environment, tissues and excretions [31].

### 2.2. Inactivated Vaccines

Heat-inactivated and formalin killed vaccines have been used in many animal models to counteract TB [32,33]. Several organisms including *M. bovis* BCG, leprosy vaccine, *Mycobacterium w* and *M. vaccae* have also been used in the form of inactivated vaccines [34,35]. A BCG vaccine killed by dehydration and rehydration followed by autoclaving before resuspending into final concentration showed no efficacy in an experimental challenge study in red deer [32]. However, HIMB is a new candidate which represents an interesting alternative to BCG, since strain survival is unlikely and deployment logistics are simpler [36]. HIMB was first prepared by heat inactivation at 80 °C for 30 min of a *M. bovis* strain isolated from a naturally infected wild boar [15]. This vaccine has yielded promising results in wild boar, both in laboratory trials [36,37] and subsequent field conditions [14,38]. In Molokai-origin wild pigs, red deer, badger and goats, HIMB vaccine has proved to have some protective effect in laboratory trials [17,18,39,40].

Another promising potential candidate alternative to BCG is MTBVAC, an attenuated *M. tuberculosis* vaccine. It is based on two independent genetic deletions in the genes *phoP* and *fadD26* [41], which encode two major virulence factors. However, the organism conserves genetic regions encoding important immunodominant antigens absent from BCG [41]. In previous studies, the SO_2_ prototype vaccine (including only the *phoP* deletion) and subsequent MTBVAC vaccine demonstrated greater immunogenicity and efficacy than BCG in mice (*Mus musculus*) and guinea pigs (*Cavia porcellus*) [42,43,44,45], as well as in rhesus macaques (*Macaca mulatta*) [46]. Moreover, both SO_2_ and MTBVAC vaccines proved to be immunogenic and effective in reducing lesion scores of TB in goats naturally exposed to *M. caprae* and *M. bovis,* respectively [47,48].

Protein subunits (Antigen (Ag) Ag85B/ESAT-6 protein, *M. bovis* culture filtrate protein (CFP), Mtb72f) along with adjuvants or mycobacterial DNA vaccines alone or in combination with DNA encoding co-stimulatory molecules such as CD80 and CD86 have been used against TB, resulting in partial protection in many animal models [49,50]. These recombinant vaccines were used in conjunction with BCG as prime boost strategy, reaching some level of protection [51,52]. There are no reports regarding the successful use of subunit/DNA/live virus vectored vaccines against TB in wildlife.

## 3. Dose and Frequency of Vaccination

Dose and frequency of vaccination are important for the type of protective immune response established and for the duration of this protection. Inferior level of protection was observed with very high dose (~10^9^) compared to medium and low dose in parenteral administration of BCG in cattle and red deer [32,53]. However, similar protection was observed in badgers when using low and high doses of BCG [54]. The optimum dose of vaccine (BCG/HIMB) is found to be higher in case of oral administration in most of the studies (10^8^–10^9^ CFU) than in case of parenteral administration (10^5^–10^6^ CFU) in both domestic and wild animals [8,15,51,55,56,57]. In cattle, combining different vaccines (i.e., BCG plus viral DNA or CFP) can induce better protection than when it is used alone [49,58,59,60], although in some cases protection was not enhanced [61]. In wild boar, Díez-Delgado et al. [62] assessed the protection and immune response achieved by homologous and heterologous regimes administering BCG and HIMB. Heterologous regimes did not improve protection over homologous regimes and showed variable results from no protection to similar protection as homologous regimes. It was concluded that homologous regimes remain the best option to vaccinate wild boar against TB. Vaccine sequences have also influenced results underlining the relevance of studying the effects of prior sensitization in the vaccination. Revaccination with BCG over a long-term interval (two years) was observed to enhance protection in cattle [50], but revaccination in young calves in a short-term interval (6 weeks) reduced the level of protection [20]. In possums, revaccination at short intervals enhanced the protective immune response; whereas, booster vaccination at longer intervals had neither detrimental nor deleterious effect on protection [63]. Oral/parenteral revaccination of BCG induced a higher protective response in case of deer and wild boar [27,32,64]. However, co-administration of BCG orally and subcutaneously did not enhance protection in cattle [56].

In oral bait delivery, possibility of underdosing or overdosing can affect the vaccine efficacy, so to avoid this, there are some methods that mitigate the overconsumption of vaccine pellets by a dominant animal like putting traps [65,66], distributing vaccine pellets more sparingly or mixing vaccine pellets with placebo pellets [67,68,69]. Even so, it has been proved that the protection induced by consumption of multiple pellets of vaccine is similar to that induced by a single oral pellet [33].

## 4. Characteristics of a Good Vaccine

An ideal vaccine should primarily have efficacy, stability and safety, limited excretion and vaccine survival, lack of interference with diagnostic tests, ease of administration as well as cost-effectiveness [70].

### 4.1. Vaccine Stability and Safety

The stability of a vaccine is an important factor mainly in oral vaccination. The stability of BCG Pasteur strain was lower compared to Danish strain [71]. The Danish strain was shown to be stable 3–5 weeks under field conditions in a forest/pasture habitat and seven weeks in the lipid matrix under room temperature conditions (21 °C, approximately) [72]. In laboratory conditions under freezing, stability of BCG vaccine was up to eight months [72]. Usually, inactivated vaccines have high stability [73]. Although the stability of HIMB vaccine has not been studied yet under field conditions, being inactivated this aspect is of lower concern [36].

Regarding safety, parenteral BCG vaccination had no unsafe reaction, except local abscesses or nodules at the inoculation point or minor adverse clinical signs [9]. Parenteral vaccination with HIMB, either subcutaneous or intramuscular delivery, produced no adverse reactions at the inoculation point in most of species [33,36,62,74], except for goats, where encapsulated abscesses with purulent content were observed in the intramuscular inoculation areas [40]. Oral vaccines (BCG/HIMB) had no adverse reactions reported [18,36,37,75,76].

### 4.2. Limited Excretion and Vaccine Survival

The excretion or shedding of BCG always increases the possibility of transmission to other animals, and it is particularly important in wildlife vaccination. However, the magnitude and duration of shedding in excretions reported was limited in almost all species studied [59,77,78]. The limited secondary transmission of BCG has occurred from vaccinated to unvaccinated in-contact white-tailed deer, but not to indirectly exposed cattle [31,79]. No BCG excretion was detected in nasal, oral, urine or fecal samples in badger and wild boar [18,36,80], including when BCG was delivered directly in the ileum [80]. On the other hand, inactivated vaccine was not reported to have persistence or shedding [18,36].

BCG was isolated at necropsy from tissues of vaccinated animals long after vaccination, depending on the post-administration time and the animal species, the route of administration, dose and type of vaccine. BCG (Danish/Pasteur strain) was recovered from orally vaccinated deer as late as three months after vaccination, while BCG persisted in subcutaneously vaccinated deer for as long as nine months in lymphoid organs, but not in the muscles [31]. Likewise, BCG could be isolated from draining lymph nodes after three months of vaccination with BCG (Pasteur) and recombinant BCG Pasteur strain in red deer [81]. BCG (Danish) was also isolated from subcutaneously vaccinated cattle and sheep [75,82], and from orally vaccinated possums and badgers [18,59,80]. However, BCG could not be found in the tissues of orally vaccinated feral swine even when examined 30 days after vaccination [83] or in wild boar when examined from 175 to 300 days post-vaccination [36]. In this regard, HIMB vaccine is not likely to persist long-term [36,40].

### 4.3. Lack of Interference with Diagnostic Tests

The SIT is recognized by the World Organisation of Animal Health (OIE) and the European Commission as the first screening test for detection of TB in cattle [84]. Other diagnostic tools including the interferon gamma (IFNγ) release assay (IGRA) were approved for use in cattle in the European Union in 2002 [Council Directive 64/432/EEC, amended by (EC) 1226/2002], received approval by the United States Department of Agriculture (USDA) in 2003 [85], and were accredited as official diagnostic tests by the Standing Committee on Agriculture in Australia in 1991. In Europe, SIT or SCITT and IGRA tests are the official diagnostic tests in the Program for the Eradication of bovine TB, which is based on the testing and slaughter of animals (Directives 64/432/EC and 78/52/EC).

It has been described that parenteral vaccination against TB interferes with official diagnostic tests, thus compromising the diagnostic strategies of official disease control and eradication programs. Using the SCITT, it was shown that 80% of calves vaccinated with BCG reacted at six months, decreasing to 10–20% at nine months after vaccination [50,86]. This interference in the diagnosis was also observed in goats vaccinated subcutaneously with BCG [48,87]. In wildlife, BCG parenteral vaccination induced a strong cellular and humoral immune response against bovine purified protein derivative (bPPD) in deer [28,32,88], white-tailed deer [57,89,90], wild boar [15], badgers [55,91,92,93] and other species [8,94]. A similar response was observed in wild boar and goats parenterally vaccinated with HIMB [15,50,95] or in goats vaccinated intranasally and subcutaneously with SO_2_ and MTBVAC, respectively [48,87]. However, oral vaccination with BCG and HIMB did not cause any diagnostic interference in the species tested [13,14,15,17,18,36,96,97,98]; even so, a recent study demonstrated that oral BCG vaccination in white-tailed deer can induce false positive skin test reactions [30].

These problems can potentially be overcome by using diagnostic tests that differentiate infected animals from vaccinated (DIVA). DIVA tests have been developed using some specific antigens of MTC, which are not expressed or secreted by BCG. Two of the antigens used in the DIVA tests are ESAT-6 and CFP-10 proteins, encoded in the RD1 region of *M. tuberculosis* and *M. bovis*, but not in BCG, which has lost this region of its genome [99,100]. The Rv3615c antigen, which is not found in the RD1 region, can also be used, but its secretion depends on the *esx-1* system located in that region [101]. An evaluation of IGRA with ESAT-6, CFP-10 and Rv3615c showed that the sensitivity was similar to the SCITT that uses bPPD and avian PPD (aPPD), while the specificity in uninfected animals was 97–99% [102]. DIVA skin test in cattle infected with *M. bovis* showed a similar sensitivity to that of SCITT, without being compromised by BCG vaccination or vaccination against paratuberculosis [103,104]. Intradermal reactivity was not observed in goats vaccinated with BCG and SO_2_ using a cocktail containing ESAT-6/CFP-10, Rv3615c and Rv3020c [87], nor was an IGRA response detected using ESAT-6 and CFP-10 in a field BCG vaccination trial with goats infected with *M. caprae* [105]. Regarding antibodies, subcutaneous BCG vaccination resulted in an increased response against lipoarabinomannan in white-tailed deer, which was not observed against CFP-10/ESAT-6 [27].

### 4.4. Delivery of Vaccine with Emphasis on Oral Bait Deployment

Delivery of vaccine is a major challenge in wildlife as far as the logistical and technical difficulties, including large target size of the population and trapping the animals, are considered [106]. Parenteral route of administration resulted in significant protection against experimental challenge or natural infection in wild boar and white-tailed deer i.e., [14,107]. Oral delivery would be the convenient choice for vaccination on a wide scale since it would be easier to deploy than an injectable vaccine and it is also a cost-effective option. In oral vaccination, BCG needs to be delivered alive in order to generate immunity—and in some species it must be protected from degradation in the stomach of the animal [33]. As an example, culture of BCG can be administered intraduodenally or intragastrically following treatment with a drug to reduce enzymatic degradation [33,108] or delivered directly in the ileum using an electronic drug delivery capsule [80]. Therefore, effective oral immunization can be successfully performed by protecting BCG via encapsulation in a lipid matrix (oral baits) and by using of selective feeding cages for deployment [26,68,89,109]. Mucosal uptake and protective immunogenicity of BCG may be enhanced using lipids in badgers [54,94] and in other species such as mice [110,111], guinea pigs [112], cattle [113] and deer [90]. A variety of carriers have been used to protect vaccines within baits intended for wildlife, such as capsules, sachets and blisters [114,115], depending on oral delivery, stability, host and age specificity. The choice of the vaccine carrier may influence the size, structure and composition of the final product, which in turn may have impact on its attractiveness and palatability [68,116].

In case of cervids, direct oral delivery as liquid was found to be effective in case of BCG in white-tailed deer [57,90] and HIMB in red deer [17] in experimental trials. Administration of BCG as oral bait with apple was used in experimental trials with white-tailed deer [57,90], while the baits with alfalfa were more palatable for red deer [69]. This type of administration was also effective in experimental and field trials in wild boar vaccinated both with BCG [14,26,64] and HIMB [14,36] included in cinnamon flavor biscuits [116], as well as in badgers and possums vaccinated with BCG embedded in peanut butter or chocolate baits, respectively [54,65,66,72,96,97]. However, there is a risk that oral baits containing BCG for wildlife may be ingested by livestock, which could result in a subsequent positive response to SIT, so it is essential to take special care with the distribution of these baits [117].

## 5. Efficacy of Vaccination

The efficacy of a vaccine is usually validated in terms of disease burden (macroscopic/microscopic lesions), presence of the organism in tissues [culture and isolation, acid fast staining, quantitative polymerase chain reaction (qPCR)] or protective immune response generated (cell mediated as well as humoral-antibody mediated). Another determining factor in the evaluation of vaccine efficacy is the challenge with MTC, in which the experimental challenge is usually more severe than the natural exposure to mycobacteria [14,15,22,36,38,54,62,65], which can lead to an undervaluation of vaccine protection [17,40].

### 5.1. Pathology and Microbiologic Examination

Presence of TB lesions is assessed by a detailed necropsy including the inspection of all relevant organs [118]. Detailed lesion scoring (macroscopic and microscopic) is a common practice to determine the degree of vaccine-induced protection in laboratory trials [15,18,23,26,27,36,39,75,89]. Total lesion prevalence was used to determine vaccine efficacy in field trials in cattle and wildlife TB studies [8,14,38,53,119,120,121]. The studies with BCG and HIMB (parenteral/oral) reduced the total disease burden, especially the thoracic lesions in goats, deer, wild boar and badgers experimentally challenged with *M. bovis* [17,18,36,48,89,91] or in field studies [14,38,65]. Disease burden was also determined with the help of tissue culture and a scoring system was developed to analyze the culture results in vaccination trials [15,17,23,33,54,105].

### 5.2. Immune Response

Measurement of cytokines produced by polyfunctional T cells has been suggested to be a potential marker for predicting vaccine efficacy [122]. Importantly, there are some implications that IFNγ, especially the ratio of IFNγ and IL-10, may act as a protection marker in vaccination with BCG/HIMB [15,17,27,123]. The IL-1β is a proinflammatory cytokine which is considered to be the main driver for the production of complement component C3 from dendritic cells and other cell types, being also the main protagonist concerned with oral vaccination [36,124]. Complement component C3 was identified as naturally associated with TB vaccine protection in wild boar [36], red deer [17,98] and zebrafish (*Danio rerio*) [39,125]. Different studies in domestic animals and wildlife have also shown how BCG induced an innate trained immune response by aerosol, oral or parenteral route [18,75,121,126]. This was also observed in goats vaccinated intranasally and subcutaneously with SO_2_ and MTBVAC, respectively [48,87]. On the other hand, parenteral administration of HIMB also induced a cellular response in cattle, goats and wild boar, but no increase in the cellular immune profile was triggered by the oral route [13,15,82,95].

With respect to antibodies, parenteral vaccination results in variable responses. Antibody response to bPPD and other MTC antigens was observed in subcutaneous vaccination with BCG in cattle [23,59,60,82], goats [48,87], sheep [75], wild boar [15], red deer [28] and badgers [92]; likewise, a humoral response was shown in parenteral vaccination with HIMB and MTBVAC in goats [48,95] and cattle [82]. In contrast, BCG/HIMB oral vaccination did not induce any antibody response either in goats, wild boar, deer or badger [15,17,18,36,57,89,95]. Previous studies of subcutaneous vaccination with BCG reported an increase in the antibody response to lipoarabinomannan enriched antigen in white-tailed deer [27], but there was no antibody response to CFP-10/ESAT-6 induced in any species since this BCG vaccine does not contain the CFP-10/ESAT-6 protein [27]. The intramuscular vaccination with HIMB in wild boar induced strong antibody response to MPB83 antigen immediately after vaccination, but not to bPPD [15].

## 6. Trials in Domestic Animals

### 6.1. Cattle

Cattle are considered the main domestic reservoir of TB worldwide and many attempts, including TB official programs based on test and cull, have tried to eradicate the disease in this species without success in some countries, i.e., United Kingdom (UK) or Spain. BCG has been used experimentally for vaccination of cattle against TB since the beginning of the 20th century [127]. Afterwards, several studies have been carried out to evaluate the efficacy of BCG vaccination in field conditions in endemic countries such as New Zealand, Chile, Mexico and Ethiopia that indicate a reduction in both the medium-term incidence and the severity of TB in vaccinated animals [8,53,119,120,128]. Many studies in experimental conditions have also been performed in cattle using different routes and vaccine candidates—BCG (several strains), HIMB, CFP and viral boosting, among others (see Table 1). In general, although full protective immunity is not induced, both lesion and bacteriological burdens are reduced to a variable extent. Factors associated with this variability include doses, inoculation route, vaccine candidate, combination with viral vectors, age at vaccination, revaccination and previous exposure to environmental mycobacteria [20,60].

### 6.2. Goat

Several studies have demonstrated the susceptibility of goats to TB [87,137,138,139], so that caprine TB is included as a notifiable disease by the OIE, although it is only actively controlled at European level when an epidemiological study shows its link with potentially infected cattle or when raw milk is used for consumption (European Regulation EC 853/2004). The effectiveness of BCG has been evaluated in goats (Table 2), observing that this vaccination does not prevent infection, although the severity of the lesions and the number of bacteria decrease, which is related to a lower transmission capacity and, therefore, spread of the mycobacterium [24,47,48,77,87]. Other experimental attenuated and inactivated vaccines such as SO_2_, MTBVAC and HIMB have been experimentally evaluated in goats (Table 2), although the preliminary results have been quite similar to those observed using BCG in terms of inability to prevent infection; however, once established, some of them have demonstrated the ability to reduce the severity of the lesions and/or bacterial load to a greater extent than BCG [47,48,74]. In this regard, work continues to improve those vaccines, testing new adjuvants or using booster strategies that combine BCG with other vaccines.

### 6.3. Sheep

There is a single study where the efficacy of BCG Danish and HIMB vaccines in sheep was assessed [75], likely because sheep were traditionally considered less susceptible to TB infection until recent years [140]. Subcutaneous administration of BCG vaccine showed considerable protection against experimental TB in lambs, measured by a reduction in the gross lesions scores and bacterial load in vaccinated animals [75]. However, a single dose of HIMB vaccine was not protective by the oral route at 10^7^ CFU/mL, since neither a reduction in the volume of gross lesions nor bacterial load in tissues were observed. The reasons may have been vaccine degradation in the lambs’ digestive system before being able to induce an effective immune response or a higher dose needed. Thus, further studies should be performed in order to evaluate the HIMB efficacy using the parenteral route in this species.

### 6.4. Pig

Even though the role of domestic pigs in the transmission of MTC has traditionally been considered limited, studies have shown that free-range domestic pigs may act as a true MTC reservoir in Mediterranean ecosystems [141,142], where they share natural resources with other domestic and wild species. To date, there is only a study where the efficacy of a vaccine was evaluated in pigs, with the aim of assessing the response of pigs with and without tonsillectomy to oral vaccination with HIMB and challenge with a virulent *M. bovis* [37]. This study did not give any evidence with regard to the effect of the presence or absence of tonsils in the lesion scores, suggesting that tonsils are not involved in the protective response to this vaccine. On the other hand, an experimental study was carried out with neonatal piglets as an animal model to test BCG efficacy in infants [143]. These animals were infected with a *M. tuberculosis* strain by aerosol route and demonstrated a similar course of TB infection and immune response to BCG compared to humans, suggesting that this model can be used for development of vaccines against TB. Additional information on pigs can be found later in this text, in the section on wild boar.

## 7. Trials in Wildlife

### 7.1. Cervids

Tuberculosis is one of the main health concerns affecting the deer farming industry and feral deer in Europe, North America, New Zealand and China [144,145]. In Europe, red deer is a known MTC maintenance host in the southwestern Iberian Peninsula and the Alpine range [145], also playing a relevant epidemiological role as long-living spillover host in New Zealand [146]. Tuberculosis vaccination in deer is consequently a field of ongoing research [9]. Most of the studies are focused on using BCG, either parenterally or orally (see Table 3), proving safety and effectiveness in red deer [28], white-tailed deer and elk (*Cervus canadensis*) [94]. Challenge experiments demonstrated similar levels of protection when BCG was administered by parenteral or oral route [57,89,90,107]. However, the vaccine efficacy does seem to be influenced by the number of doses administered, being higher in white-tailed deer that received two subcutaneous doses of 10^7^ CFU against those that received only one [27]. Oral HIMB vaccination also induced a partial reduction of the TB lesion score in red deer challenged with a *M. bovis* field strain without interfering with the in vivo diagnosis [17]. Nevertheless, these results could be underestimated due to high challenge doses via the intra-tracheal route. Further experiments with this new vaccine candidate using more realistic challenge routes and higher sample sizes are necessary.

### 7.2. Wild Boar

There is scientific evidence that wild boar plays a crucial role in the maintenance of MTC in the Iberian Mediterranean ecosystems [145,147,148]. Nevertheless, TB was reported in this species in many European countries [145], Asia [149], North Africa [150] and South America [151]. BCG and HIMB vaccines, by parenteral or oral route, have shown in this species a significant protection in different laboratory challenge trials (see Table 4). HIMB vaccination also achieved a progressive reduction in the TB lesions prevalence in farmed wild boar or in field conditions, both at low and high prevalence settings [14,38]. Hence, this vaccination strategy may contribute, along with other tools, to reach TB control in wild boar. In field conditions, the model used with oral vaccine baits was focused on piglets because they are less likely to be infected [14]. Further studies are needed for evaluating whether this vaccination scheme could reduce the TB lesion prevalence in adult boars. The effectiveness of HIMB was also proved in Molokai wild pigs, where the oral vaccination induced a modest degree of infection containment [16].

### 7.3. Badger

European badgers are recognized TB maintenance hosts in the UK and the Republic of Ireland (ROI) [152,153,154,155,156]. Novel studies suggest that badgers may be a potential reservoir of MTC infection also in Atlantic Spain [25,157] and France, especially in hot-spot areas where prevalence in cattle remains high [158,159]. Vaccination of badgers has been proposed as a long-term control strategy for TB in addition to culling in UK and ROI [160]. Experimental studies have demonstrated that vaccination with BCG vaccine is protective in badgers using the oral, parenteral or oral bait routes [18,54,91,97] (Table 5). At the moment, only BCG is permitted for intramuscular administration to badgers in the UK since 2010, and there are limitations for its delivery in the nature despite of the fact that different studies have shown its efficacy in field conditions [65,66]. Oral administration of HIMB vaccine conferred protection against experimental TB in badgers [18], appearing to be a promising oral vaccine candidate for badgers.

### 7.4. Brushtail Possum

The introduced Australian brushtail possum is a reservoir host for *M. bovis* in New Zealand [161]. TB emerged in this species in the late 1960s. Tuberculosis in possums is usually lethal, and most animals die within a few months of infection. Control has been achieved by a variety of methods, including major periodic reductions in possum density (nowadays typically > 90%) at about 5-year intervals using aerial poisoning and lesser reductions, usually at 1–2 year intervals, using ground-based trapping or poisoning [162]. As well as in badgers, vaccination of possums has been proposed as a TB control measure. BCG (Pasteur or Danish) has been the vaccine of choice in almost all trials, and both strains have induced protection using different routes—conjunctival, intranasal aerosol, oral, intragastric, intraduodenal, subcutaneous—and doses [163,164], even in field conditions [121,165,166] (Table 6). Some products such as ranitidine have shown to reduce gastric acidity and improved the efficacy of intragastrically administered BCG [108].

### 7.5. African Buffalo

Tuberculosis is endemic in wildlife and domestic animals in South Africa. The first confirmed case in an African buffalo (*Syncerus caffer*) in this country occurred within the Hluhluwe–iMfolozi Park in 1986. Afterwards, several cases have been reported in National Parks, game Reserves or private farms, in which buffalos have been implicated in cattle infection [170,171]. Several proposals have been put forward to prevent TB from spreading, including fencing, intensive culling and vaccination. In this regard, efficacy of subcutaneously administered BCG was assessed, which did not yield any protection in this species (Table 1). Different factors such as age of animals, route of vaccination or challenge dose, among others, were considered. Despite those results, vaccination is considered as a promising strategy and an integral part of TB control in South Africa pending future experiments [171].

### 7.6. Ferrets

Feral ferrets (*Mustela furo*) are scavengers that can also become infected with *M. bovis*. In New Zealand, ferrets are considered as spill-over hosts for TB. However, they could become potential maintenance hosts of TB if factors such as population density exceed the estimated threshold for disease persistence [172]. As a possible control measure, oral and subcutaneous routes of vaccination with BCG Pasteur reduced the severity of the disease following experimental infection with *M. bovis* [173,174].

## 8. Conclusions and Future Research Priorities

Tuberculosis vaccination does not induce full protective immunity but moderates the severity of the infection and of onward transmission. Thus, vaccination of domestic species and wildlife is a strategy that should be seriously considered. Official programs for the control and eradication of animal TB are extremely expensive and only target the bovine species. It should be also considered that the nonspecific effects of mycobacterial vaccination can be even a more important reason for the widespread use of this type of vaccines, especially in its inactivated version [175,176,177]. The advantages of the inactivated vaccine with respect to live BCG include: (i) it does not generate adverse reactions in laboratory or farm trials, (ii) there is no strain survival in vaccinated hosts or in the field, (iii) it can be delivered orally with no evidence to date of sensitizing ruminants to SIT (i.e., not giving false positive animals) and (iv) it is stable in storage and at high environmental temperatures [13,14]. However, it has also limitations compared to BCG, i.e., nowadays legislation does not permit its use and fewer experiments have been performed, thus future research is needed. Currently, vaccination against TB is not permitted for cattle in Europe (Directive 78/52/EC), due to its incompatibility with official diagnostic tests (Directive 64/432/EC), although is considered in some countries such as the UK [178]. In this sense, the development and use of DIVA tests will be essential in countries or regions with low TB prevalence that export domestic animals and hunting species or their products, with the aim of differentiating infected animals from those vaccinated with BCG; in countries with a high prevalence of TB, where vaccination is unlikely to induce complete protection against the disease, its use without DIVA tests in species not subjected to official eradication programs could be useful to reduce the spread of *M. bovis* to cattle.

In high-prevalence regions it has been shown that the contact between different domestic species, raised in extensive systems, and the wild MTC reservoirs favors the circulation and maintenance of mycobacteria in the environment [179,180]. Therefore, interest in the development and use of TB vaccines in wildlife has grown and it is perceived as an alternative, which has also been favored by a better understanding of the immune response to the disease, the development of DIVA reagents and the greater investment in the development of vaccines for humans and domestic animals. As already mentioned, experimental trials of vaccination against TB in wildlife have increased considerably in recent years, obtaining promising vaccine efficacy results. Moreover, the use of new animal and challenge models for vaccine research have increased in the last years, such as those performed in zebrafish, which will shorten experiment duration and reduce costs, thereby expediting effective research [39,125]. However, the protection induced by vaccines against the disease could decrease through time, being important to improve such protection by eliciting a longer immune response through application of better adjuvants or by revaccination. For this, it would be necessary to increase the time of vaccination efficacy and facilitate the administration of the vaccine, having to explore in greater depth its deployment in oral baits, the efficacy and stability of the oral bait in field conditions, the survival of the vaccine in tissues and its possible excretion or transmission to the environment.

In all these situations, vaccination should never be implemented alone, but should be included in a strategic plan for integrated control of TB under a ‘’one health’’ perspective (human-animal-environment), which also includes other measures such as breeding cattle for resistance, improved biosecurity on farms to avoid or decrease contact between domestic and wild animals or population control of wildlife to avoid overabundance that may favor the maintenance of the disease. All these measures must be implemented and evaluated to mitigate the risk of transmission of MTC in a multi-host environment. For this purpose, novel research has focused on improving good efficacy of vaccine, and at the same time ensuring its administration, safety and stability in the environment.

In addition, taking into account that funding resources invested in TB programs will be likely decreased in coming years due to the disruption of new emerging diseases worldwide [181], public and private (farming and hunting) sectors will have to adapt and adjust animal management strategies accordingly.

## Figures and Tables

**Table 1:** Description of the vaccination assays in bovids.

Species	Type of Vaccine	Route and Dose of Vaccine	Challenge	Method of Evaluation of Protective Efficacy	Result	Reference
Cattle	BCG	SC (100 mg/10 mL), 1 dose–several doses	Natural challenge	Skin test	Restricted vaccine efficacy	[127]
Cattle	BCG (Pasteur)	SC (6 × 10^4^ or 6 × 10^6^ CFU), 1 dose	800 CFU *M. bovis* ITC	TBL, culture, IGRA, skin test, antibodies	Vaccine efficacy at both doses	[23]
Cattle	BCG (Pasteur)	SC (10^6^ CFU), 1 dose at 8 h or 6 weeks of age SC (10^6^ CFU), 2 doses (6 weeks interval)	1.5 × 10^3^ CFU *M. bovis* ITC	TBL, culture, IGRA, cytokines, skin test, antibodies	Better vaccine efficacy at birth Non-environmental mycobacteria sensitization Revaccination contraindicated	[20]
Cattle	BCG (Pasteur) DNA (Hsp 65, Hsp 70) DNA+BCG	DNA: intradermal and IM (1 mg/mL), 1 dose BCG: SC (1 × 10^6^), 1 dose Combined DNA prime + BCG boost	1.5 × 10^3^ CFU *M. bovis* ITC	TBL, histology, culture, IGRA, cytokine assay, ELISPOT, skin test	Vaccine efficacy, better combined	[58]
Cattle	BCG (Pasteur)	Oral (10^8^ CFU), 1 pellet Oral (10^8^ CFU), 10 pellets SC (10^6^ CFU), 1 dose	5 × 10^3^ CFU *M. bovis* ITC	TBL, histology, culture, IGRA, cytokine assay (IL-2), skin test	Similar vaccine efficacy for SC and oral (10 pellets) routes	[113]
Cattle	BCG (Pasteur)	SC (10^6^ CFU), 1 dose	5 × 10^3^ CFU *M. bovis* ITC	TBL, histology, culture, IGRA, ELISPOT, skin test	Vaccine efficacy	[129]
Cattle	BCG (Pasteur) CFP BCG + CFP	CFP: SC, 2 doses BCG: SC (10^6^), 1 dose Combined BCG + CFP, 1 dose	5 × 10^3^ CFU *M. bovis* ITC	TBL, culture, IGRA, skin test, antibodies	Vaccine efficacy, better combined	[59]
Cattle	Combined DNA prime *M. tuberculosis* BCG (Tokyo) boost	Combined DNA prime: IM (1500 μg), 1 dose BCG: IM (1 × 10^6^), 1 dose Combined DNA prime + BCG boost	1 × 10^7^ CFU *M. bovis* ITC	TBL, histology, culture, IGRA, skin test, antibodies	Vaccine efficacy, better combined	[60]
Cattle	BCG (Danish/ Pasteur)	BCG Danish (fresh culture): SC (10^6^), 1 dose BCG Pasteur (fresh culture): SC (10^6^), 1 dose BCG Danish (freeze-dried culture): SC (1–4 × 10^6^), 1 dose	5 × 10^3^ CFU *M. bovis* ITC	TBL, histology, culture, IGRA, skin test	Similar vaccine efficacy	[130]
Cattle	BCG (Pasteur)	Oral (10^9^ CFU), 1 dose each SC (10^6^ CFU), 1 dose Oral + SC	10^3^ CFU *M. bovis* ITC	TBL, histology, culture, IGRA, skin test	Similar vaccine efficacy by both routes, not enhanced by co-administration	[56]
Cattle	BCG + MVA85A	SC (10^6^ CFU), 1 dose	2 × 10^3^ CFU *M. bovis* ITC	TBL, histology, culture, IGRA, ELISPOT	Vaccine efficacy with viral boosting	[49]
Cattle	BCG (Danish)	SC (10^6^ CFU), 1 dose	Natural challenge	TBL, culture, IGRA, skin test	Vaccine efficacy in field conditions	[119]
Cattle	BCG (Danish)	SC (10^6^ CFU), 1 dose	Natural challenge	IGRA, skin test	Vaccine efficacy (lower excretion) in field conditions	[128]
Cattle	BCG (Danish) DeltaRD1	SC (10^6^ CFU), 1 dose each	10^3^ CFU *M. bovis* Aerosol	TBL, histology, culture, lung radiography	Vaccine efficacy	[131]
Cattle	BCG (Danish)	Oral (10^6^, 10^7^, 10^8^ CFU), 1 dose each SC (10^6^ CFU), 1 dose	5 × 10^3^ CFU *M. bovis* ITC	TBL, culture, IGRA, skin test	Vaccine efficacy at high oral dose or SC	[51]
Cattle	BCG (Danish/Pasteur)	SC (2 × 10^6^ CFU), 1 dose each	3 × 10^3^ CFU *M. bovis* ITC	TBL, histology, culture, IGRA, ELISPOT	Vaccine efficacy	[21]
Cattle	BCG (Danish) CFP/Chitin/Gel 01 CFP/Emulsigen/ Pam_3_CSK_4_	BCG: Oral (10^8^, 2 × 10^7^ CFU), 1 dose each BCG: SC (1 × 10^6^ CFU), 1 dose CFP/Chitin/Gel 01: IN (0.4 mg), 1 dose CFP/Emulsigen/ Pam_3_CSK_4_: SC (0.4 and 0.25 mg), 1 dose Combined BCG (oral) + CFP/Chitin (IN) Combined BCG (oral) + CFP/ Emulsigen (SC)	5 × 10^3^ CFU *M. bovis* ITC	TBL, histology, culture, IGRA, skin test	Vaccine efficacy not enhanced by co-administration of mycobacterial protein vaccines	[61]
Cattle	*M. bovis* Δmce2 double deletion mutant BCG (Pasteur)	*M. bovis* Δmce2: SC (10^6^ CFU), 1 dose BCG (Pasteur): SC (10^6^ CFU), 1 dose	10^6^ CFU *M. bovis* ITC	TBL, histology, culture, IGRA, cytokines in PBMC, skin test	Vaccine efficacy. *M. bovis* Δmce2 conferred better protection than BCG	[132]
Cattle	BCG (Danish)	SC: High dose 1 × 10^6^ to 4 × 10^6^ CFU, 1 dose SC: low dose 1 × 10^5^ to 4 × 10^5^ CFU, 1 dose	5 × 10^3^ CFU *M. bovis* ITC	TBL, histology, culture, IGRA, skin test	Similar vaccine efficacy at both doses	[133]
Cattle	BCG (Phipps)	SC (10^6^ CFU), 1 dose	5 × 10^5^ CFU *M. bovis*, IN	TBL, histology, IGRA	Vaccine efficacy with CFP boosting	[134]
Cattle	BCG (Danish) TB BioBead (Ag85A+ESAT-6) CFP	BCG: SC (2–8 × 10^5^ CFU), 1 dose Revaccination: BCG: SC (2–8 × 10^5^ CFU) TB BioBead: SC (200 μg) CFP: SC (400 μg)	5 × 10^3^ CFU *M. bovis* ITC	TBL, histology, culture, IGRA, skin test, antibodies	Revaccination with BCG boosts protection	[50]
Cattle	BCG (Danish) Adenovirus (Ad) 85A	BCG: SC (10^6^ CFU), 1 dose BCG: EB (10^6^ CFU), 1 dose Combined BCG: SC (5 × 10^5^ CFU)+EB (5 × 10^5^ CFU), 1 dose Combined BCG (SC, 10^6^ CFU) + Ad85A (EB, 2 × 10^9^ PFU), 1 dose	2 × 10^3^ CFU *M. bovis* EB	TBL, histology, culture, IGRA, ELISPOT	Better vaccine efficacy in BCG/BCG and BCG/Ad85 protocol	[22]
Cattle	HIMB	Oral/IM (10^6^–10^7^ CFU), 1 dose	No challenge	Skin test, IGRA	Cellular immune response profile not increased by oral route	[13]
Cattle	BCG (Danish) HIMB *M. bovis* BCG formalin-inactivated	BCG: SC (2 × 10^6^ CFU), 1 dose HIMB: SC (1 × 10^7^ CFU), 1 dose Formalin-inactivated: SC, 1 dose	2 × 10^8^ CFU BCG Danish intranodular	Culture, skin test, IGRA, antibodies	HIMB vaccine clearly immunogenic	[82]
Cattle	BCG (Danish)	Oral (1 × 10^8^ CFU), 1 dose Oral (2 × 10^7^ CFU), 1 dose SC (3 × 10^5^ CFU), 1 dose	Natural challenge	TBL, culture, skin test	Vaccine efficacy at high oral dose or SC in field conditions	[8]
Cattle	BCG (Danish)	SC (3 × 10^5^ BCG), 1 dose	Natural challenge	TBL, culture	Vaccine efficacy at low dose in field conditions	[53]
Cattle	BCG (Danish)	SC (1–4 × 10^6^ CFU), 1 dose	Natural challenge	TBL, histology, culture, IGRA, skin test	Vaccine efficacy in field conditions	[120]
Cattle	BCG (Danish)	In vitro assay (1 × 10^5^ cells/well) In vivo: Aerosol (1 × 10^8^ CFU), 1 dose	No challenge	Antibodies, PBMCs, cytometry	Induction of innate cell-mediated immune response	[126]
Cattle	BCG (Danish) + MVA85A	SC (BCG: 2 × 10^6^ CFU), 1 dose SC (combined BCG: 2 × 10^6^ CFU + Ad85A: 10^9^ PFU), 1 dose	2 × 10^7^ CFU BCG Intranodally	TBL, culture, IGRA, ELISPOT, antibodies	Induction of cellular and humoral immune response	[52]
Zebu (*Bos indicus*)	BCG	SC (0.1 mg), 2 doses	1 mg *M. bovis*, oral	TBL, histology, culture, skin test	Vaccine efficacy	[135]
African buffalo (*Syncerus caffer*)	BCG (Pasteur)	SC, 2 doses (first 3.2 × 10^7^, booster 4.4 × 10^7^)	1 × 10^3^ CFU and 6 × 10^2^ CFU *M. bovis*, ITC	TBL, histology, culture, IGRA, skin test	No vaccine efficacy	[136]

All studies included a non-vaccinated group (control). BCG—Bacillus Calmette–Guérin; HIMB—heat-inactivated *Mycobacterium bovis* vaccine; CFP—*M. bovis* culture filtrate vaccine; *M. tuberculosis*—*Mycobacterium tuberculosis*; SC—subcutaneous; IM—intramuscular; IN—intranasal; ITC—intratracheal; CFU—colony forming units; PFU—plaque forming units; EB—endobronchial; LST—lymphocyte stimulation test; ELISPOT—enzyme-linked immunospot assay; TBL—tuberculosis-like macroscopic lesions; IGRA—interferon gamma (IFNγ) release assay; IL—interleukin; PBMC—peripheral blood mononuclear cells.

**Table 2:** Description of the vaccination assays in goat.

Type of Vaccine	Route and Dose of Vaccine	Challenge	Method of Evaluation of Protective Efficacy	Result	Reference
BCG (Danish) MVA85A	SC (BCG: 5 × 10^5^ CFU), 1 dose SC (combined BCG: 5 × 10^5^ CFU + Ad85A: 10^9^ PFU), 1 dose	1.5 × 10^3^ CFU *M*. *caprae*, EB	TBL, culture, skin test, IGRA, serology	Cellular and humoral immune response. BCG-AdAg85A reduced pulmonary TBL compared to BCG	[24]
BCG (Danish) SO_2_	SC (BCG: 1–4 × 10^5^ CFU), 1 dose SC/IN (SO_2_: 10^5^ CFU), 1 dose	No challenge	Skin test, IGRA	Skin test and IGRA response. DIVA antigens could be used to differentiate BCG and SO_2_ vaccinated	[87]
BCG (Danish)	SC (5 × 10^5^ CFU), 1 dose	No challenge	TBL, culture, IGRA	IGRA response. No lack of biological safety, negligible environment and public health and local adverse reactions	[77]
BCG (Danish)	SC (10^5^ CFU), 1 dose	Natural challenge *M. caprae*	TBL, culture, histology, PCR, serology	Great reduction of TBL	[105]
BCG (Danish) SO_2_	SC (BCG: 1–4 × 10^5^ CFU), 1 dose SC (SO_2_: 10^5^ CFU), 1 dose	Natural challenge *M. caprae*	TBL, culture, skin test, IGRA	SO_2_ vaccinated had the lowest lesion and culture scores	[47]
HIMB	Oral/IM (6 × 10^7^ CFU), 2 doses	No challenge	Skin test, IGRA, serology	No positivity to the SIT or IGRA test in orally vaccinated	[95]
HIMB	IM (10^7^ CFU), 2 doses	Natural challenge *M. caprae*	TBL, culture, skin test, IGRA, serology	Reduction of TBL, but not significantly	[40]
BCG (Danish) HIMB	SC (BCG: 5 × 10^5^ CFU), 1 dose SC (HIMB: 10^7^ CFU), 1 dose IM (HIMB: 10^7^ CFU), 1 dose	2 × 10^4^ CFU *M*. *caprae*, EB	TBL, culture, IGRA, serology	Similar protection to BCG in reduction of TB lesions and bacterial load	[74]
BCG (Danish) MTBVAC	SC (BCG: 2–8 × 10^5^ CFU), 1 dose SC (MTBVAC: 5 × 10^5^ CFU), 1 dose	Natural challenge *M. caprae*	TBL, culture, skin test, IGRA, serology	Immunogenicity and reduced severity of TB pathology in both vaccines	[48]

BCG—Bacillus Calmette–Guérin; HIMB—heat-inactivated *Mycobacterium bovis* vaccine; *M. caprae*—*Mycobacterium caprae*; SC—subcutaneous; IN—intranasal; IM—intramuscular; CFU—colony forming units; EB—endobronchial; TBL—tuberculosis-like macroscopic lesions; IGRA—interferon gamma (IFNγ) release assay; PCR—polymerase chain reaction; DIVA—diagnostic tests to differentiate vaccinated from infected animals.

**Table 3:** Description of the vaccination assays in cervids.

Species	Type of Vaccine	Route and Dose of Vaccine	Challenge	Method of Evaluation of Protective Efficacy	Result	Reference
Red deer	BCG Pasteur (live, dead, with or without adjuvant)	SC (5 × 10^7^ CFU), 2 doses	No challenge	Skin test, LST	Immunoprotective response in live BCG group	[28]
Red deer	BCG Pasteur (live, lyophilized)	SC (5 × 10^4^, 5 × 10^7^, 5 × 10^8^ CFU), 2 doses	2–5 × 10^2^ CFU *M. bovis*, IT	Skin test, TBL, histology, culture, LST	Protection in low and medium dose, less at high dose	[32]
	BCG Pasteur (live + DXM, dead)	SC (2.5 × 10^6^ CFU)/ IT (5 × 10^7^ CFU), 2 doses			No vaccine efficacy	
Red deer	BCG (Pasteur)	SC (5 × 10^6^ CFU), 2 doses	2–5 × 10^2^ CFU *M. bovis* *, IT	Skin test, TBL, culture, LST, antibodies	Vaccine efficacy	[88]
Elk	BCG (Pasteur)	SC (10^7^ CFU), 2 doses	No challenge	PBMC proliferation assay, flow cytometry and ELISA	Antibody response, proliferation of lymphocytes and macrophages	[94]
White-tailed deer	BCG (Pasteur)	SC (10^7^ CFU), 1/2 doses	300 CFU *M. bovis*, IT	TBL, histology	Vaccine efficacy, higher with 2 doses	[27]
White-tailed deer	BCG (Danish)	Bait (10^9^ CFU)/oral (10^9^ CFU)/SC (10^6^ CFU), 1 dose	228 CFU *M. bovis*, IT	TBL, culture, histology, lymphocyte proliferation, MAPIA, IGRA	Vaccine efficacy by both administration routes	[90]
White-tailed deer	BCG (Pasteur/ Danish)	SC (10^7^ CFU), 1 dose	990 CFU *M. bovis*, IT	TBL, culture	Vaccine efficacy, more with Danish. Vaccine persistence	[89]
White-tailed deer	BCG (Danish)	Bait (10^9^ CFU) /oral (1,9 × 10^8^) /SC (3,4 × 10^6^ CFU), 1 dose	228 CFU *M. bovis*, IT	MAPIA, Rapid test, IB, antibodies, culture	Vaccine efficacy	[57]
White-tailed deer	BCG (Danish)	Oral (10^8^ CFU), 1 dose	300 CFU *M. bovis*, IT	TBL, histology, culture, IGRA, antibodies	Vaccine efficacy	[107]
Red deer	HIMB	Oral (6 × 10^6^ CFU), 1 dose	No challenge	Antibodies, C3, IFNγ. IL-1β	C3 response in serum	[98]
Red deer	HIMB BCG (Danish)	Oral (10^7^ CFU), 2 doses Oral (10^8^ CFU), 2 doses	10^6^ CFU *M. bovis*, ITC	TBL, culture, antibodies, IGRA, IFNγ, ILs, C3	Partial efficacy of both vaccines. Too high dose of *M. bovis*	[17]
White-tailed deer	BCG (Danish)	Liquid oral (10^8^ CFU/10^10^ CFU), 1 dose	No challenge	Skin test	Greater false positives with a higher vaccine dose	[30]

All studies included a non-vaccinated group (control); * Infection at different times upon vaccination (6, 26 and 52 weeks post-vaccination); BCG—Bacillus Calmette–Guérin; HIMB—heat-inactivated *Mycobacterium bovis* vaccine; DXM—dexamethasone; SC—subcutaneous; IT—intratonsillar, ITC—intratracheal; CFU—colony forming units; PBMC—peripheral blood mononuclear cells; LST—lymphocyte stimulation test; TBL—tuberculosis-like macroscopic lesions; IGRA—interferon gamma (IFNγ) release assay; MAPIA—multi-antigen printing immunoassay; IB—immunoblot; IL—interleukins; C3—complement factor 3.

**Table 4:** Description of the vaccination assays in wild swine.

Species	Type of Vaccine	Route and Dose of Vaccine	Challenge	Method of Evaluation of Protective Efficacy	Result	Reference
Wild boar	BCG	Oral bait (15–30 × 10^5^ CFU), 1 dose	10^4^ CFU *M. bovis*, OF	TBL, IL-4, C3, IFNγ, MUT	Upregulation of immunomodulatory genes	[26]
Wild boar	BCG HIMB	Oral/IM (BCG: 10^8^ CFU; HIMB: 6 × 10^6^ CFU), 2 doses	10^6^ CFU *M. bovis*, OF	TBL, culture, IGRA, antibodies, C3, MUT	Efficacy with both vaccines by both administration routes	[15]
Wild boar	HIMB	Oral bait (10^7^ CFU), 2 doses	10^5^ CFU *M. bovis*, OF	TBL, culture, antibodies, IGRA, IL-1β, C3, MUT	Vaccine efficacy	[36]
Wild boar	BCG HIMB	Oral bait (BCG: 5.2–7.6 × 10^6^ CFU; HIMB: 10^7^ CFU)	No challenge	Survival of BCG by culture, excretion of *M. bovis* by PCR	No adverse reaction, survival or excretion with any vaccine	[37]
Wild boar	BCG	Oral bait, (10^6^ CFU/bait), 2 doses	10^5^ CFU *M. bovis*, OF	TBL, culture, antibodies, IGRA, IL-1β, C3, MUT	Vaccine efficacy	[64]
Wild boar	HIMB *	IM (6 × 10^6^ CFU), 2 doses	No challenge	TBL, antibodies	Vaccine efficacy	[38]
Wild boar	BCG HIMB *	Oral bait, (15–30 baits of 10^5^ CFU)	No challenge	TBL, antibodies	Vaccine efficacy	[14]
Wild boar	Combined BCG + BCG Combined HIMB + HIMB Combined BCG + HIMB Combined HIMB + BCG	Oral (BCG: 10^6^ CFU; HIMB: 10^7^ CFU), 2 doses	10^5^ CFU *M. bovis*, OF	TBL, culture, IGRA, antibodies, C3, MUT	Homologous regimes are the best option to vaccination	[62]
Molokai-origin wild pigs	HIMB	Oral (10^7^ CFU), 2 doses	10^6^ CFU *M. bovis*, Oral	TBL, histology, culture	Partial vaccine efficacy	[16]

All studies included a non-vaccinated group (control). BCG—Bacillus Calmette–Guérin (all studies used the Danish strain); HIMB—heat-inactivated *Mycobacterium bovis* vaccine; IM—intramuscular; CFU—colony forming units; OF—oropharyngeal; *—Assays performed under field conditions, so infective dose cannot be determined; TBL—tuberculosis-like macroscopic lesions; IGRA—interferon gamma (IFNγ) release assay; ILs—interleukins; C3—Complement factor 3; MUT—methylmalonyl-CoA mutase.

**Table 5:** Description of the vaccination assays in badgers.

Type of Vaccine	Route and Dose of Vaccine	Challenge	Method of Evaluation of Protective Efficacy	Result	Reference
BCG (Danish)	SC/IM (16–22 × 10^7^ CFU/ 4–7 × 10^5^ CFU), 2 doses	No challenge	Culture, LST, ELISPOT	Induction of cell-mediated immune response	[55]
BCG (Danish)	Oral bait (10^8^ CFU), 1 dose	10^4^ CFU *M. bovis*, EB	TBL, histology, culture, IGRA, ELISPOT	Vaccine efficacy	[96]
BCG (Danish)	IM (3.2–5.4 × 10^6^ CFU), 1 dose	2.6–4.8 × 10^3^ CFU *M. bovis*, EB Natural challenge	TBL, histology, culture, IGRA, antibodies	Vaccine efficacy in experimental study, but not in field conditions	[92]
BCG (Danish)	IM (3.3 × 10^5^–5.4 × 10^6^ CFU), 1 dose	2.6–4.8 × 10^3^ CFU *M. bovis*, EB	TBL, histology, culture, ELISPOT	Vaccine efficacy	[91]
BCG (Danish)	IM (2–8 × 10^6^ CFU), several doses	Natural challenge	Culture, IGRA, antibodies	Reduction of infection risk by *M. bovis*	[93]
BCG (Danish/Pasteur)	Oral bait (both, 10^8^ CFU), 1 dose	6 × 10^3^ CFU *M. bovis*, EB	TBL, histology, culture, ELISPOT	Vaccine efficacy with both vaccine types	[97]
BCG (Danish)	Oral bait (9.6 × 10^6^–3.2 × 10^8^ CFU) IT (9.3 × 10^7^ CFU), 1 dose	0.98–1.85 × 10^3^ CFU *M. bovis*, EB	TBL, histology, culture, ELISPOT, antibodies	Vaccine efficacy at low and high doses	[54]
BCG (Danish)	Oral bait (10^8^ CFU), 2 doses	Natural challenge	TBL, histology, culture, antibodies	Vaccine efficacy	[65]
BCG (Danish)	Oral bait (10^8^ CFU), 2 doses	Natural challenge	TBL, histology, culture, antibodies	Vaccine efficacy	[66]
BCG (Danish) HIMB	Oral (live BCG: 10^8^ CFU; HIMB: 10^7^ CFU), 1 dose	10^3^ CFU *M. bovis* EB	Necropsy, culture, skin test, IGRA, serology, molecular methods, MRI analysis	Protection of HIMB similar to BCG by reducing TBL	[18]

All studies included a non-vaccinated group (control). BCG—Bacillus Calmette–Guérin; HIMB—heat-inactivated *Mycobacterium bovis* vaccine; SC—subcutaneous; IM—intramuscular; IT—intratonsillar; CFU—colony forming units; EB—endobronchial; LST—lymphocyte stimulation test; ELISPOT—enzyme-linked immunospot assay; TBL—tuberculosis-like macroscopic lesions; IGRA—interferon gamma (IFNγ) release assay.

**Table 6:** Description of the vaccination assays in possums.

Type of Vaccine	Route and Dose of Vaccine	Challenge	Method of Evaluation of Protective Efficacy	Result	Reference
BCG (Pasteur)	IN aerosol (4 × 10^6^ CFU), 1 dose Oral (3 × 10^8^ CFU), 1 dose SC (1 × 10^6^ CFU), 1 dose	400 CFU *M. bovis*, ITC	Lymphocyte proliferation assay, TBL, histology, culture	Vaccine efficacy IN and SC administered	[19]
BCG (Pasteur)	SC (1 × 10^6^ CFU), 1 dose Intragastric (1 × 10^8^ CFU), 1 dose Intraduodenal (1 × 10^8^ CFU), 1 dose	20 CFU *M. bovis*, ITC	Lymphocyte proliferation assay, TBL, histology, culture	Vaccine efficacy	[163]
BCG (Pasteur)	IN aerosol (5–6.5 × 10^6^ CFU), 1 dose	*M. bovis*, ITC, 2 (28 and 78 CFU), 6 (78 CFU) and 12 (50 CFU) months post-vaccination	Lymphocyte proliferation assay, TBL, histology, culture	Vaccine efficacy, better when challenge was performed 2 months post-vaccination	[167]
BCG (Pasteur)	Conjunctival (5 × 10^6^ CFU) and IN aerosol (5 × 10^6^ CFU), 1 dose Revaccination every 4–5 months	100 CFU *M. bovis*, ITC	Lymphocyte proliferation assay, TBL, histology, culture	Vaccine efficacy	[29]
BCG (Pasteur)	Conjunctival and IN aerosol, 1, 2 or 12 doses (1 × 10^8^ CFU)	100 CFU *M. bovis*, EB	Lymphocyte proliferation assay, TBL, histology, culture	Vaccine efficacy, better 12 doses	[63]
BCG (Pasteur) Heat-killed *M. vaccae* BCG + heat-killed *M. vaccae*	Conjunctival and IN aerosol (2 × 10^6^ CFU), 1 dose Heat-killed *M. vaccae* (3 × 10^9^ mycobacteria), 1 dose	80 CFU *M. bovis*, ITC	Lymphocyte proliferation assay, TBL, histology, culture	Vaccine efficacy, better combined	[168]
BCG (Pasteur)	Oral (2 × 10^8^ CFU), 1 dose Oral bait (1 × 10^8^ CFU), 1 dose	10–20 *M. bovis* bacilli/animal, aerosol	Lymphocyte proliferation assay, TBL, culture	Similar vaccine efficacy	[71]
BCG (Pasteur)	Conjunctival (2.5 × 10^5^ CFU), 1 dose	100 CFU *M. bovis*, ITC	Lymphocyte proliferation assay, TBL, histology, culture	Vaccine efficacy	[169]
BCG (Pasteur)	BCG intragastrically (10^8^ CFU) + 75 mg ranitidine, 1 dose Ranitidine, 1 dose BCG, 1 dose	100 CFU *M. bovis*, ITC	Lymphocyte proliferation assay, TBL, culture	Vaccine efficacy enhanced with ranitidine	[108]
BCG (Pasteur)	Oral bait (1 × 10^8^ CFU or 5–10 × 10^8^ CFU), 1 dose	No challenge	Lymphocyte proliferation assay, culture	Vaccine survival in associated lymph nodes and excretion in feces up to 7 days	[59]
BCG (Danish/Pasteur)	Oral: 10 pellets heat-inactivated BCG Pasteur (10^8^ bacilli) + revaccination 15 weeks later with 1 pellet live BCG Pasteur (10^7^ CFU) 1 pellet live BCG Pasteur (10^7^ CFU), 1 dose 1 pellet live BCG Danish (10^7^ CFU), 1 dose 10 pellets live BCG Pasteur (10^8^ CFU), 1 dose SC live BCG Pasteur (10^6^ CFU), 1 dose	10–20 *M. bovis*, aerosol	Lymphocyte proliferation assay, TBL, culture	Similar vaccine efficacy, slightly better SC	[33]
BCG (Danish)	Oral bait (1 × 10^8^ CFU)	10–20 *M. bovis* bacilli/animal, aerosol	TBL, culture	Lipid baits with 10% chocolate are more palatable Vaccine efficacy	[72]
BCG (Danish)	Oral (1 × 10^7^ CFU), 1 dose Revaccination at 6 months	Natural challenge	Lymphocyte proliferation assay, TBL, culture	Vaccine efficacy in field conditions	[165]
BCG (Danish)	Oral (1 × 10^7^ CFU), 1 dose	High dose: 100 CFU *M. bovis*, SC, 2 doses Low dose: 10 CFU *M. bovis*, SC, 2 doses	TBL, culture	Sustained protection for 12 months in field conditions	[166]
BCG (Danish)	Oral (1 × 10^8^ CFU), 1 dose	Natural challenge	Lymphocyte proliferation assay, TBL, culture	Vaccine efficacy in field conditions	[121]

All studies included a non-vaccinated group (control); BCG—Bacillus Calmette–Guérin; *M. bovis*—*Mycobacterium bovis*; *M. vaccae*—*Mycobacterium vaccae;* IN—intranasal; SC—subcutaneous; ITC—intratracheal; EB—endobronchial; CFU—colony forming units; TBL—tuberculosis-like macroscopic lesions.
